# Highly Selective Adsorption of ^99^TcO_4_^−^/ReO_4_^−^ by a Novel Polyamide-Functionalized Polyacrylamide Polymer Material

**DOI:** 10.3390/toxics10100630

**Published:** 2022-10-21

**Authors:** Ben Qin, Yanqin Hu, Meiying Xie, Liyan Xue, Chunfa Liao, Fan Yang

**Affiliations:** 1 Faculty of Materials, Metallurgy and Chemistry, Jiangxi University of Science and Technology, Ganzhou 341000, China; 2 Xiamen Institute of Rare Earth Materials, Haixi Institute, Chinese Academy of Sciences, Xiamen 361021, China; 3 Fujian Province Joint Innovation Key Laboratory of Fuel and Materials in Clean Nuclear Energy System, Fujian Institute of Research on the Structure of Matter, Chinese Academy of Sciences, Fuzhou 350002, China; 4 Fujian Science & Technology Innovation Laboratory for Optoelectronic Information of China, Fuzhou 350108, China; 5 Sichuan Jcc Rare Earth Matals New Material Co., Ltd., Chengdu 610213, China

**Keywords:** polyacrylamide, polyamidation, ^99^TcO_4_^−^/ReO_4_^−^ removal, polymer adsorption material

## Abstract

The treatment of radioactive wastewater is one of the major problems in the current research. With the development of nuclear energy, the efficient removal of ^99^TcO_4_^−^ in radioactive wastewater has attracted the attention of countries all over the world. In this study, a novel functional polyamide polymer p-(Amide)-PAM was synthesized by the two-step method. The experimental results show that p-(Amide)-PAM has good adsorptive properties for ^99^TcO_4_^−^/ReO_4_^−^ and has good selectivity in the nitric acid system. The kinetics of the reaction of p-(Amide)-PAM with ^99^TcO_4_^−^/ReO_4_^−^ was studied. The results show that p-(Amide)-PAM has a fast adsorption rate for ^99^TcO_4_^−^/ReO_4_^−^, the saturated adsorption capacity reaches 346.02 mg/g, and the material has good reusability. This new polyamide-functionalized polyacrylamide polymer material has good application prospects in the removal of ^99^TcO_4_^−^ from radioactive wastewater.

## 1. Introduction

With the advent of international “carbon peaking and carbon neutrality”, advanced nuclear energy has attracted the attention of countries all over the world, but with the development of advanced nuclear energy, the treatment of technetium 99 (^99^Tc) in nuclear waste has become a major problem [1,2]. A large amount of radioactive wastewater is produced in the process of nuclear waste material storage [3]. ^99^Tc is the most potentially problematic radionuclide in radioactive wastewater because it decays by emitting β particles, and its half-life is as long as 2.13 × 10^5^ years [4,5]. ^99^Tc is a super hydrophilic radionuclide that mainly exists in radioactive wastewater in the form of ^99^TcO_4_^−^. ^99^TcO_4_^−^ has high water solubility (11.3 mol/L, 293.15 K) and almost no complexation properties, resulting in rapid migration in the environment and thus making it a dangerous radioactive pollutant [6,7]. Therefore, it is of great significance to remove ^99^TcO_4_^−^ from radioactive wastewater.

It has always been a difficult point for researchers to remove and capture ^99^TcO_4_^−^ in radioactive wastewater under the condition of strong acid, strong alkali, and a large number of anions [8]. The current research methods for removing and capturing ^99^TcO_4_^−^ in radioactive wastewater can be divided into solvent extraction and solid-phase extraction [9,10,11,12]. Compared with solvent extraction, solid phase extraction has a faster extraction rate, better reusability, simpler operation, and easier separation [13]. Therefore, solid phase extraction is a method that is widely used and studied in practice, and solid-phase extraction is a commonly used adsorbent. The most studied adsorbents are metal-organic frameworks (MOFs) and covalent organic frameworks (COFs). They have a high specific surface area and many pores, so they have high a saturated adsorption capacity for ^99^TcO_4_^−^, but they are insufficient for the treatment of ^99^TcO_4_^−^ in environments with high acidity and acidity [14,15,16,17,18,19]. Ion exchange resins are other polymer materials that have been widely studied. Such resins can effectively remove ^99^TcO_4_^−^ from radioactive wastewater by anion substitution and can maintain a good adsorption performance under highly acidic and alkaline conditions. Ion exchange resins have simple operation and a high recovery rate, but the removal efficiency and selectivity become very poor in the presence of a large number of competitive anions (SO_4_^2^^−^, NO_3_^−^, Cl^−^, etc.) [20,21]. Some people have also studied the removal of ^99^TcO_4_^−^ by natural zeolites. They modified natural zeolites with cationic surfactants to convert the negative charges of the surface framework of zeolites into positive charges, thus enhancing the affinity for ^99^TcO_4_^−^. However, the saturated adsorption content of ^99^TcO_4_^−^ on zeolites is generally low [22,23]. By aminating the functional groups of chitosan, researchers removed ^99^TcO_4_^−^ under high-acidity conditions, but chitosan has the same disadvantages as zeolite [24,25]. High-molecular-weight polymers are also adsorbents that can effectively remove ^99^TcO_4_^−^ from radioactive wastewater. Many polymers have been developed and used to remove ^99^TcO_4_^−^ in radioactive wastewater [26,27,28,29,30]. After polymers are modified by polyamidation, the polymer can selectively remove ^99^TcO_4_^−^ by electrostatic interaction or hydrogen bonding [28,31,32]. Polyacrylamide (PAM) is a widely used polymer that is often used in biomedicine, sewage treatment, and other fields. As an adsorbent, PAM can effectively remove anions in wastewater. Therefore, the removal of ^99^TcO_4_^−^ from radioactive wastewater using PAM is also an important research topic [33,34,35].

^99^TcO_4_^−^ has strong radioactivity, and ReO_4_^−^, which has similar physical and chemical properties, is used to replace ^99^TcO_4_^−^ in experiments [36]. In this study, a new type of polyamide-functionalized polyacrylamide polymer material was prepared by a two-step method, and its adsorption effect on ^99^TcO_4_^−^/ReO_4_^−^ was studied. In a certain range of pH values, p-(Amide)-PAM has a good effect on the removal of ^99^TcO_4_^−^/ReO_4_^−^. Under the influence of different competitive anions, p-(Amide)-PAM has excellent selectivity for ^99^TcO_4_^−^/ReO_4_^−^. The adsorption kinetics of p-(Amide)-PAM for ^99^TcO_4_^−^/ReO_4_^−^ was studied, and the material has a fast adsorption rate and high saturated adsorption capacity (346.02 mg/g). This study provides a new material design direction for the treatment of ^99^TcO_4_^−^ in radioactive wastewater.

## 2. Materials and Methods

### 2.1. Materials and Reagents

Polyacrylamide ((C_3_H_5_NO)_n_, PAM, cationic; molecular weight 1800) was purchased from Beijing Huawei Ruike Chemical Co., Ltd, Beijing, China. Ammonium perrhenate (NH_4_ReO_4_, ≥ 99.99%) was purchased from Shanghai Dibo Biotechnology Co., Ltd, Shanghai, China. Ethylenediamine (C_2_H_8_N_2_, analytical purity) was provided by Shanghai Aladdin Limited Chemical Reagent Co., Ltd, Shanghai, China. Ammonia (NH_3_▪H_2_O), nitric acid (HNO_3_), and dimethylformamide (C_3_H_7_NO, DMF, 0.945 g/mL) were all analytically pure and were purchased from Sinopharm Chemical Reagent Co., Ltd, Beijing, China. The ultra-pure water (18.2 m Ω cm) used in this experiment was obtained from a Direct-Q3UV purification system (Research Water Purification Technology Co., Ltd, Xiamen City, Fujian Province, China).

### 2.2. Synthesis of p-(Amide)-PAM

**Figure 1:**
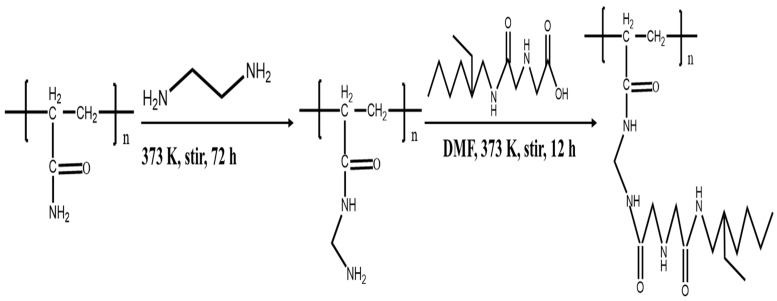
Preparation flow chart of p-(Amide)-PAM.

The synthesis process of p-(Amide)-PAM is shown in Figure 1. Step 1: According to published articles, the p-(Amide)-PAM preparation process was as follows [37,38]: 4.2 g PAM was added to 30 mL of ethylenediamine at 373 K stirred and heated for 72 h, and the resulting product was washed 3 times with ethanol. Then, the resulting solid product was placed in a 353 K vacuum oven for 48 h, and the solid is named N-PAM.

Step 2: Amide acid was synthesized according to the literature [39]. The amide acid was mixed with N-PAM in 30 mL DMF. The mixture was heated and stirred at 373 K for 12 h. Then the product was washed 3 times with ethanol before dried at 353 K for 48h, resulting in a yellow solid powder named p-(Amide)-PAM.

### 2.3. Characterization of PAM, N-PAM, and p-(Amide)-PAM

The elemental (C, H, O, and N) contents of PAM, N-PAM, and p-(Amide)-PAM were determined by elemental analysis (Vario El Cube, Germany) and X-ray photoelectron spectroscopy (XPS, K-alpha+, U.K.). The surface morphologies of PAM, N-PAM, and p-(Amide)-PAM were observed by field emission scanning electron microscopy (Apreo S LoVac, Czech Republic). The surface functional groups in the range of 4000-500 cm^−1^ were obtained by Fourier transform infrared spectroscopy (FT-IR, Nicolet iS 50, USA). The specific surface area and porosity of N-PAM and p-(Amide)-PAM were measured by an automatic specific surface area and porosity analyzer (Quantachrome Autosorb IQ, USA). FT-IR and XPS were used to analyze p-(Amide)-PAM before and after adsorption of ReO_4_^−^ to explore its potential adsorption mechanism.

### 2.4. Batch Adsorption Experiments

The original solution of ReO_4_^−^ with a concentration of 1000 mg/L was prepared with ammonium perrhenate, and other desired concentrations of ReO_4_^−^ were prepared by further dilution of this solution. All adsorption experiments were carried out in 15 mL centrifuge tubes on a constant temperature shaker with a rotational speed of 250 rpm. The pH value of the solution was adjusted with 0.1 M HNO_3_ and NH_3_▪H_2_O. The initial concentration of ReO_4_^−^ was 0–1000 mg/L, the contact time was 0–15 h, the pH value was 1–14, and the initial pH was 5.5. After adsorption, the liquid was filtered through a 0.22 μm nylon filter. The initial metal concentration and residual metal concentrations of the samples were determined by inductively coupled plasma optical emission spectrometry (ICP-OES, Ultima2, France).

The adsorption capacity of the adsorbent was calculated by *Q_e_* (mg/g) and removal rate (*R*) was calculated by the following formulas (Formulas (1) and (2)):

(1) $$Q_{e}=(C_{0}-C_{e})\times \frac{V}{m}$$

(2) $$R\%=\frac{C_{0}-C_{e}}{C_{0}}\times 100\%$$

where *C*_0_ and *C_e_* are the initial concentration and equilibrium concentration (mg/L) of ReO_4_^−^, *Q_e_* is the adsorption capacity at equilibrium (mg/g), *V* is the volume of the aqueous phase (L), and *m* is the mass of the adsorbent (g).

The Langmuir model and Freundlich model were used to fit the isothermal adsorption data and are expressed by Formulas (3) and (4) [40]:

(3) $$\frac{C_{e}}{Q_{e}}=\frac{1}{K_{L}Q_{\max}}+\frac{C_{e}}{Q_{\max}}$$

(4) $$\ln Q_{e}=\frac{1}{n}\ln C_{e}+\ln K_{F}$$

where *C_e_* refers to the concentration of ReO_4_^−^ at equilibrium (mg/L), *Q_max_* is the theoretical maximum adsorption capacity (mg/g), *K_L_* is the Langmuir constant (L/mg), and *K_F_* (mg/g (L/mg)1/n) and 1/n are Freundlich constants.

The pseudo-first-order kinetic model and pseudo-second-order kinetic model of adsorption kinetics are expressed by Formula (5) and Formula (6), respectively [41]:

(5) $$\ln(Q_{e}-Q_{t})=\ln(Q_{e}){-k}_{1}t$$

(6) $$\frac{t}{Q_{t}}=\frac{t}{Q_{e}}+\frac{1}{k_{2}Q_{e}^{2}}$$

where *C_e_* refers to the concentration of ReO_4_^−^ at equilibrium (mg/L); *Q_e_* and *Q_t_* are the adsorption capacity (mg/g) of ReO_4_^−^ at equilibrium and at time *t* (min), respectively; and *k*_1_ and *k*_2_ are the pseudo-first-order and pseudo-second-order kinetic model constants.

## 3. Results and Discussion

### 3.1. Characterizations of PAM, N-PAM, and p-(Amide)-PAM

**Figure 2:**
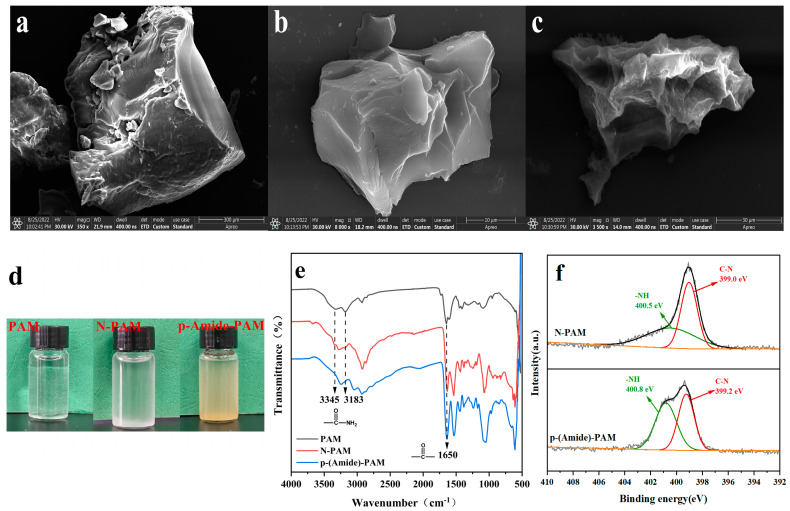
SEM images of PAM (**a**), N-PAM (**b**), and p-(Amide)-PAM (**c**); dispersion of PAM, N-PAM, and p-(Amide)-PAM in water (**d**); FT-IR spectrum of PAM, N-PAM, and p-(Amide)-PAM (**e**); and XPS N 1s spectrum of N-PAM and p-(Amide)-PAM (**f**).

The scanning electron microscopy pictures of PAM (Figure 2a), N-PAM (Figure 2b), and p-(Amide)-PAM (Figure 2c) are shown in the figure and their elemental content is shown in Table 1. It can be seen from the diagram that the morphology of PAM is large particles that are relatively regular; N-PAM and p-(Amide)-PAM show irregular small particles after the reaction. By comparing the N_2_ adsorption-desorption isotherms of N-PAM (Figure S1a) and p-(Amide)-PAM (Figure S1b), it can be seen that the specific surface area of p-(Amide)-PAM is 90 times higher than the specific surface area of N-PAM, and the pores of p(Amide)-PAM (Figure S1c) are microporous. Figure 2d shows the dispersion of PAM, N-PAM, and p-(Amide)-PAM in water. PAM dissolved in water and formed a hydrogel while N-PAM and p-(Amide)-PAM were insoluble in water. In the FT-IR spectrum (Figure 2e), the characteristic peaks of -NH_2_ at 3345 and 3183 cm^−1^ are greatly weakened after the reaction, indicating that most of the -NH_2_ is involved in the reaction process. The peak at 1650 cm^−1^ is characteristic of C=O, and the intensity of the peak of synthesized p-(Amide)-PAM increases substantially, which indicates that the functional modification of N-PAM by amide acid was successful. In the XPS wide scan spectrum of N-PAM (Figure S2a) and p-(Amide)-PAM (Figure S2b), there are N1s, C1s, and O1s spectrum. In the XPS N 1s spectrum (Figure 2f), the -NH (400.8 eV) peak of p-(Amide)-PAM is significantly increased, indicating a significant increase in the number of -NH groups on p-(Amide)-PAM. The binding energies of the modified -NH and C-N increase from 400.5 and 399.0 to 400.8 and 399.2 eV, respectively, indicating that the modification reaction took place on the amino group [42,43,44]. These results further indicate that the functional modification of N-PAM by amide acid was successful.

### 3.2. Adsorption Experiment of ReO_4_^−^ by p-(Amide)-PAM

#### 3.2.1. Influence of Different Molar Reaction Ratios

**Figure 3:**
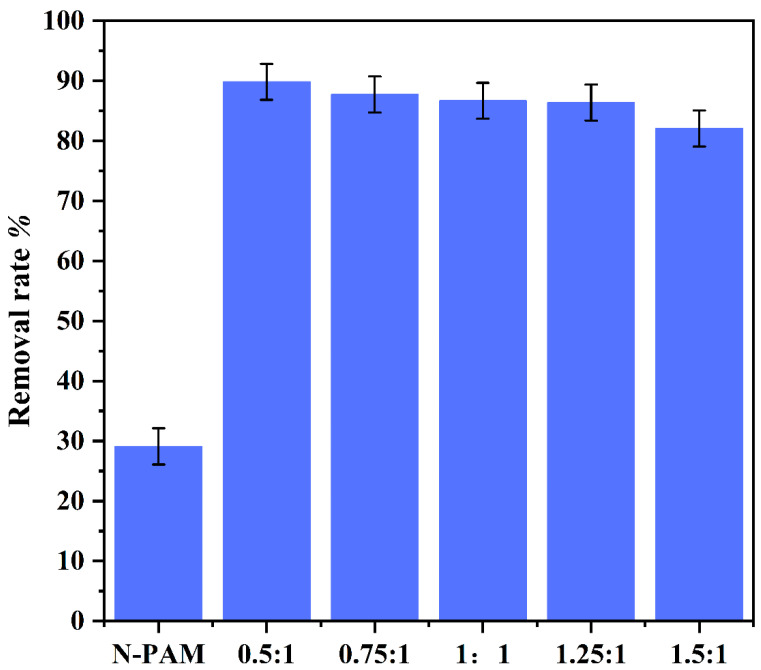
Removal rate of ReO_4_^−^ by p-(Amide)-PAM generated by different reaction molar ratios. The dosage of the absorbent was 1 g/L, pH was 5.5, time was 240 min, initial concentration of ReO_4_^−^ was 100 mg/L, and the temperature was 298.15 K.

The effect of the amount of amide acid on the removal of ReO_4_^−^ in the synthesis of p-(Amide)-PAM was investigated. Five groups of samples were prepared with the molar ratios of amide acid to N-PAM of 0.5:1, 0.75:1, 1:1, 1.25:1, and 1.5:1. In Figure 3, the removal rate of ReO_4_^−^ by the intermediate N-PAM is only 29%. The removal rate of p-(Amide)-PAM significantly improves after the functionalization of polyamides. When the molar ratio of the reaction is 0.5:1, the removal rate of ReO_4_^−^ is the highest (up to 90%). With the increase in the amide acid molar ratio, the removal rate of ReO_4_^−^ by p-(Amide)-PAM decreases. This may be due to the N-H functional groups of the adsorbents occupying the adsorption sites of ReO_4_^−^. These results show that the addition of amide acid has an effect on the removal of ReO_4_^−^ by p-(Amide)-PAM, especially when the molar amount of amide acid is greater than that of N-PAM.

#### 3.2.2. Effect of Initial pH

**Figure 4:**
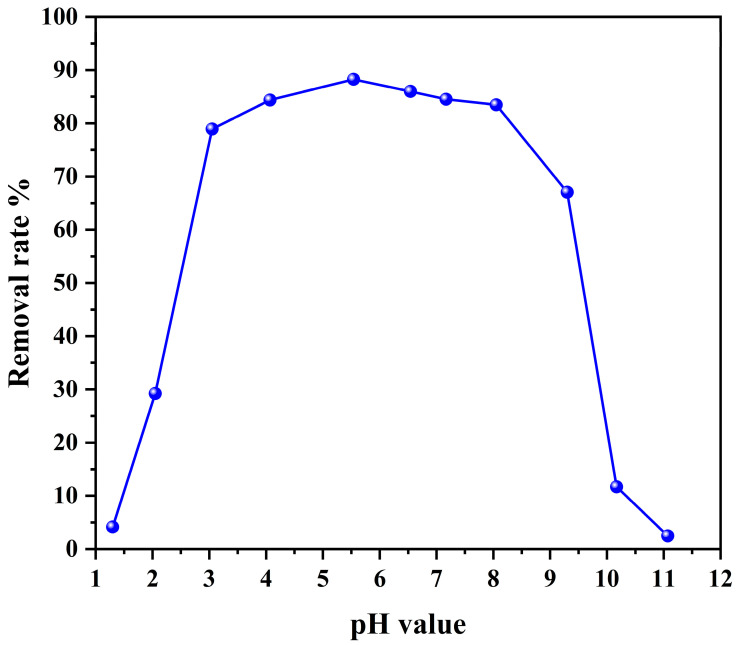
The pH effect on the removal efficiency for ReO_4_^−^ by p-(Amide)-PAM. The dosage of the absorbent was 1 g/L, time was 240 min, initial concentration of ReO_4_^−^ was 100 mg/L, and the temperature was 298.15 K.

The effect of p-(Amide)-PAM on ReO_4_^−^ removal under acid-base conditions was explored. In this study, adsorption experiments under different pH conditions were performed, as shown in Figure 4. As the pH increases from 1.3 to 4.0, the removal rate of ReO_4_^−^ by p-(Amide)-PAM gradually increases (from 4% to 80%). When the pH is in the range of 4.0 to 8.0, the removal rate of ReO_4_^−^ by p-(Amide)-PAM is more than 80%. When the pH is 5.5, the maximum removal rate is 88%. From pH 8.0 to 11.0, the removal rate of ReO_4_^−^ by p-(Amide)-PAM decreases gradually, and the removal rate is only 3% when the pH is 11.0. In the case of low pH, the removal rate of ReO_4_^−^ by p-(Amide)-PAM is low, which may be due to the high concentration of NO_3_^−^ and the lack of protonation of -NH on p-(Amide)-PAM. As the pH increases, -NH can protonate to produce a positive charge, resulting in electrostatic interactions with ReO_4_^−^. In an alkaline environment, OH^−^ in the aqueous phase will be attracted by protonated -NH, which occupies the adsorption sites, resulting in a decline in the adsorption effect of ReO_4_^−^. Compared to amino triazole-modified microcrystalline cellulose microsphere and ionic liquid-MIMDIDOA, p-(Amide)-PAM can efficiently remove ReO_4_^−^ over a wide range of pH values [42,45].

#### 3.2.3. Influence of Competitive Anions

**Figure 5:**
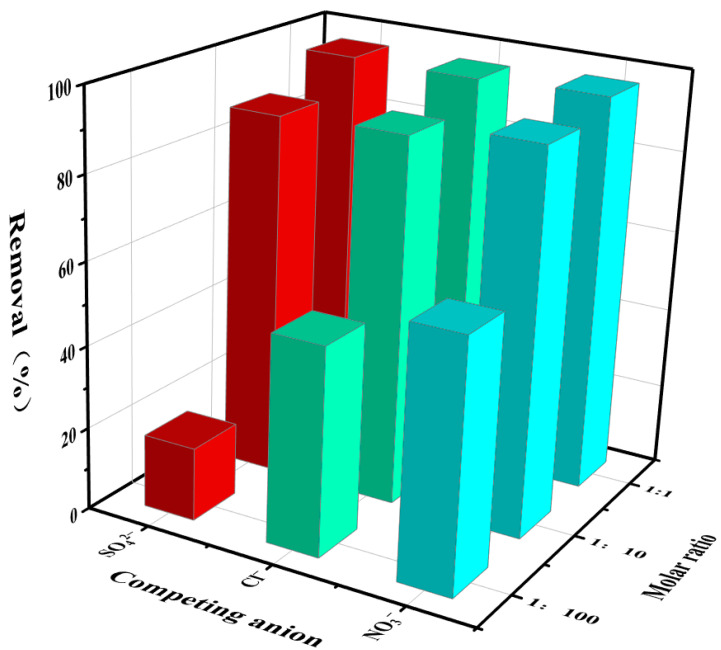
Effect of competing anions on the removal percentage of ReO_4_^−^ by p-(Amide)-PAM. The dosage of the absorbent was 4 g/L, pH was 5.5, time was 240 min, initial concentration of ReO_4_^−^ was 10 mg/L, and the temperature was 298.15 K.

There are a large number of competitive anions (NO_3_^−^, Cl^−^, SO_4_^2−^, etc.) in radioactive wastewater, which will adversely affect the adsorption of ReO_4_^−^. As shown in Figure 5, when the molar ratio of ReO_4_^−^ to competing anions is 1:1, the removal rate of ReO_4_^−^ by p-(Amide)-PAM is 95.7–96.7%. When the molar ratio of ReO_4_^−^ to competitive anions is 1:100, the removal rate of ReO_4_^−^ by p-(Amide)-PAM still reaches 60%. The selectivity of p-(Amide)-PAM may be attributable to its hydrophobic surface and ReO_4_^−^ has a relatively low hydration energy (−170 kJ/mol). Compared with other anions such as NO_3_^−^ (−275 kJ/mol) and Cl^−^ (−340 kJ/mol), the hydrophobic surface of p-(Amide)-PAM more easily adsorbs ReO_4_^−^. In addition, the negative charge of SO_4_^2−^ (−1080 kJ/mol) is higher than that of ReO_4_^−^, and SO_4_^2−^ is a more favorable compound for adsorption via electrostatic interactions [28]. According to the research report of existing adsorbents, in the anionic system of SO_4_^2−^ or Cl^−^, the adsorbent is more effective in removing ^99^TcO_4_^−^/ReO_4_^−^ from radioactive wastewater, but it is interesting that in this work, the effect is better under the system of NO_3_^−^.

#### 3.2.4. Adsorption Isotherm

**Figure 6:**
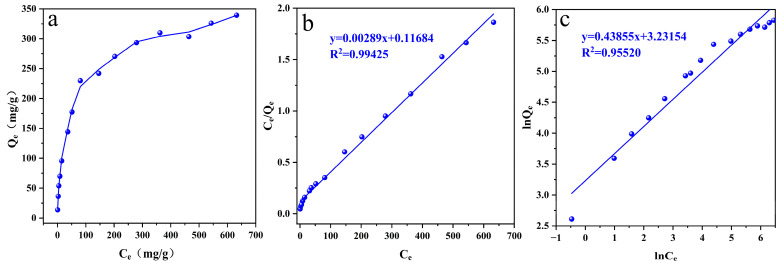
Adsorption isotherm of ReO_4_^−^ by p-(Amide)-PAM (**a**): fitting the Langmuir model (**b**) and fitting the Freundlich model (**c**). The dosage of the absorbent was 1 g/L, pH was 5.5, time was 12 h, initial concentration of ReO_4_^−^ was 0–1000 mg/L, and the temperature was 298.15 K.

In order to explore the adsorption performance, the adsorption isotherm of ReO_4_^−^ by p-(Amide)-PAM (Figure 6a) was tested, and the adsorption isotherm data were fitted by the Langmuir model (Figure 6b, Table 2) and Freundlich model (Figure 6c, Table 2). Through the comparison of the two models, the adsorption of ReO_4_^−^ by p-(Amide)-PAM is more consistent with the Langmuir model (R^2^ = 0.99452), indicating that the adsorption is monolayer chemisorption on a homogeneous surface. The results show that the saturated adsorption capacity of p-(Amide)-PAM for ReO_4_^−^ is as high as 346.02 mg/g. Compared with the reported adsorbent materials (Table 3), the saturated adsorption capacity of p-(Amide)-PAM exceeds that of most adsorbent materials.

#### 3.2.5. Adsorption Kinetics

**Figure 7:**
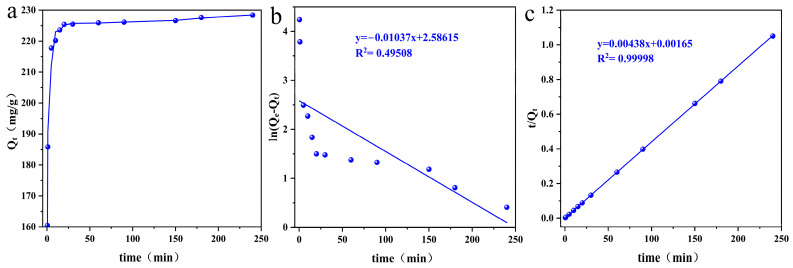
Adsorption kinetics of p-(Amide)-PAM for ReO_4_^−^ (**a**): fitting pseudo-first-order kinetic model (**b**) and fitting pseudo-second-order kinetic model (**c**). The dosage of the absorbent was 1 g/L, pH was 5.5, time was 0–240 min, initial concentration of ReO_4_^−^ was 300 mg/L, and the temperature was 298.15 K.

The adsorption properties were further explored, and the adsorption kinetics of ReO_4_^−^ by p-(Amide)-PAM were determined (Figure 7a); the data were fitted by a pseudo-first-order kinetic model (Figure 7b, Table 4) and pseudo-second-order kinetic model (Figure 7c, Table 4). Figure 7a shows that the adsorption rate is fast during the initial stage of adsorption, more than 80% of ReO_4_^−^ is adsorbed in approximately 60 s, and adsorption equilibrium is gradually reached after 120 s. In the first stage, the rapid adsorption process is mainly controlled by physical diffusion, and ReO_4_^−^ quickly occupies the effective adsorption sites. The slow adsorption in the second stage mainly depends on chemical adsorption, which continues until the adsorption equilibrium is reached. Comparing the pseudo-first-order kinetic model and the pseudo-second-order kinetic model, the adsorption of ReO_4_^−^ by p-(Amide)-PAM is more consistent with the pseudo-second-order kinetic model, which indicates that the adsorption process is controlled by chemical adsorption such as surface complexation and metal coprecipitation [49]. The adsorption rate and equilibrium time of adsorbents are important factors for evaluating the performance of adsorbents, where a fast rate and short equilibrium time correspond to a good performance. Therefore, p-(Amide)-PAM has good prospects for the removal of ^99^TcO_4_^−^ in radioactive wastewater.

### 3.3. Sorption Mechanism

**Figure 8:**
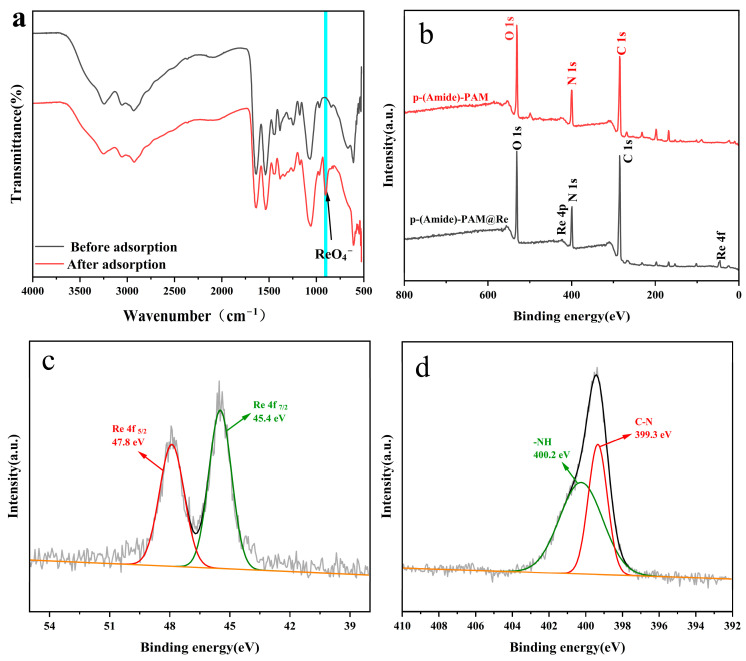
FT-IR spectra (**a**) and XPS wide scan spectrum (**b**) of p-(Amide)-PAM and p-(Amide)-PAM@Re; XPS Re 4f spectrum (**c**) and XPS N 1s spectrum (**d**) of p-(Amide)-PAM@Re.

The sorption mechanism of ReO_4_^−^ by p-(Amide)-PAM was studied by XPS and FT-IR. The comparison of the FT-IR spectra (Figure 8a) before and after the adsorption of ReO_4_^−^ by p-(Amide)-PAM shows a new peak at 903 cm^−1^ after adsorption, which corresponds to the stretching vibration of Re-O formed by electrostatic interactions between ReO_4_^−^ and protonated -NH groups [25,27]. Comparing the XPS (Figure 8b) patterns before and after the adsorption of ReO_4_^−^ by p-(Amide)-PAM, the peaks of Re 4f_5/2_ (47.8 eV) and Re 4f_7/2_ (45.4 eV) in Re 4f of p-(Amide)-PAM@Re (Figure 8c) are visible, which indicates that the removed ReO_4_^−^ still exists in the form of ions. In the N 1s pattern of p-(Amide)-PAM@Re (Figure 8d), the binding energies of -NH (400.2 eV) and C-N (399.3 eV) have changed, indicating electrostatic interactions between amino groups and ReO_4_^−^ after protonation.

## 4. Conclusions

In this study, the polymer material p-(Amide)-PAM with polyamide functionalization was successfully synthesized. The results show that p-(Amide)-PAM has good selective adsorption properties for ^99^TcO_4_^−^/ ReO_4_^−^. The removal of ^99^TcO_4_^−^/ReO_4_^−^ by p-(Amide)-PAM has a relatively wide pH window (3.0-8.0) and maintains an excellent adsorption performance. P-(Amide)-PAM maintains good selectivity in environments with a large number of competitive anions (NO_3_^−^, Cl^−^, SO_4_^2−^), and was best under the NO_3_^−^ system. P-(Amide)-PAM has a fast adsorption rate (adsorption equilibrium after 120 s) and high saturated adsorption capacity (346.02 mg/g) for ^99^TcO_4_^−^/ReO_4_^−^. The predominant ^99^TcO_4_^−^/ReO_4_^−^ sorption mechanism by p-(Amide)-PAM was the electrostatic interaction of amino groups with ^99^TcO_4_^−^/ReO_4_. These results all indicate that p-(Amide)-PAM has good application prospects in the rapid and deep removal of ^99^TcO_4_^−^/ReO_4_^−^ from radioactive wastewater.

## Figures and Tables

**Table 1:** The elemental content of PAM, N-PAM, and p-(Amide)-PAM.

Elemental	PAM	N-PAM	p-(Amide)-PAM
C [%]	45.140	42.667	39.926
H [%]	7.580	8.670	7.559
O [%]	30.577	29.696	36.562
N [%]	16.517	18.994	15.508

**Table 2:** Fitting parameters of the Langmuir model and Freundlich model.

Models	Parameters	p-(Amide)-PAM
Langmuir	*K_L_*	0.0247
	*Q*_max_ (mg/g)	346.02
	R^2^	0.99452
Freundlich	*K_F_*	25.319
	*n*	2.28
	R^2^	0.95520

**Table 3:** Adsorption capacity of ^99^TcO_4_^−^/ReO_4_^−^ by different adsorption materials.

Adsorbent	Adsorption Capacity (mg/g)	References
p-(Amide)-PAM	346.02	This work
GO-DEADIBA	140.82	[32]
SCU-100	541	[15]
SCU-101	217	[46]
SCU-102	291	[17]
SCU-103	318	[47]
Ag-TPPE	251	[19]
ZJU-X6	507	[18]
3-ATAR	146.4	[42]
DNOA–GO–CS	90.33	[25]
CSN	222	[48]
SCU-CPN-4	437	[30]

**Table 4:** Fitting parameters of the pseudo-first-order model and pseudo-second-order model.

Models	Parameters	p-(Amide)-PAM
Pseudo-first-order	*k*_1_	0.01037
	*Q_e1_*(mg/g)	13.279
	R^2^	0.49508
Pseudo-second-order	*k*_2_	0.0116
	*Q_e2_*(mg/g)	228.311
	R^2^	0.99998

## Data Availability

Not applicable.

## Supplementary Materials

The following supporting information can be downloaded at: https://www.mdpi.com/article/10.3390/toxics10100630/s1, Figure S1a: N_2_ adsorption-desorption isotherms of the N-PAM, Figure S1b: N_2_ adsorption-desorption isotherms of the p-(Amide)-PAM, Figure S1c: pore size distribution of N-PAM and p-(Amide)-PAM; Figure S2a: XPS wide scan spectrum of N-PAM, Figure S2b: XPS wide scan spectrum of p-(Amide)-PAM.
