# Supplementary material for: Highly Selective Adsorption of 99TcO4−/ReO4− by a Novel Polyamide-Functionalized Polyacrylamide Polymer Material
Source: Toxics. 2022 Oct 21;10(10):630. doi: 10.3390/toxics10100630 (PMC9608480; doi:10.3390/toxics10100630)
Supplement: Supplementary file 1 [file toxics-10-00630-s001.zip › toxics-1966370-supplementary.pdf]

## Supplementary Materials

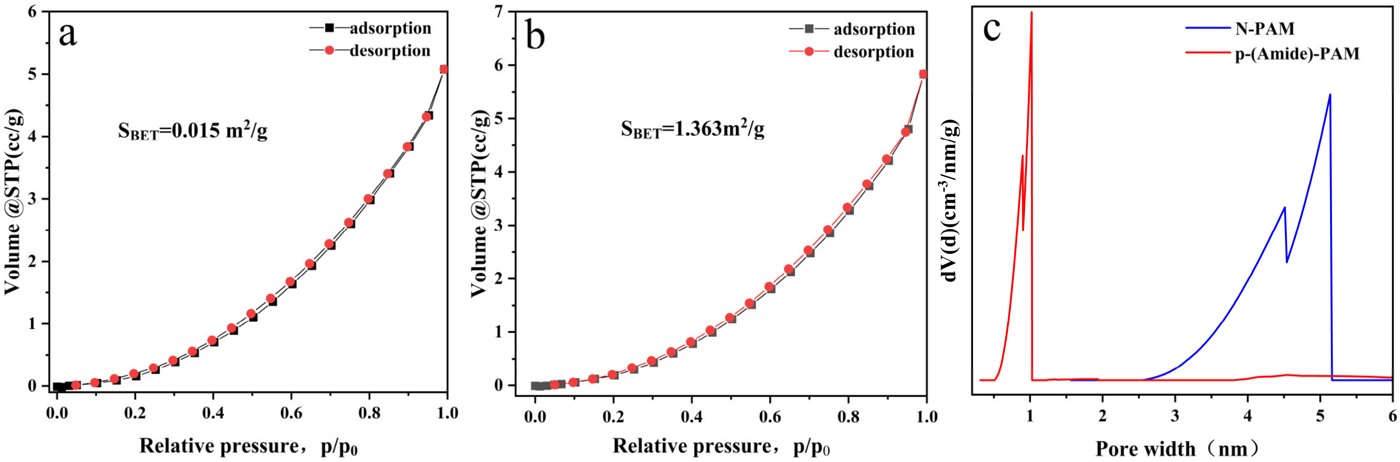


**Figure S1.** $N_2$ adsorption-desorption isotherms of the N-PAM **(a)** and p-(Amide)-PAM **(b)**, pore size distribution of N-PAM and p-(Amide)-PAM **(c)**.

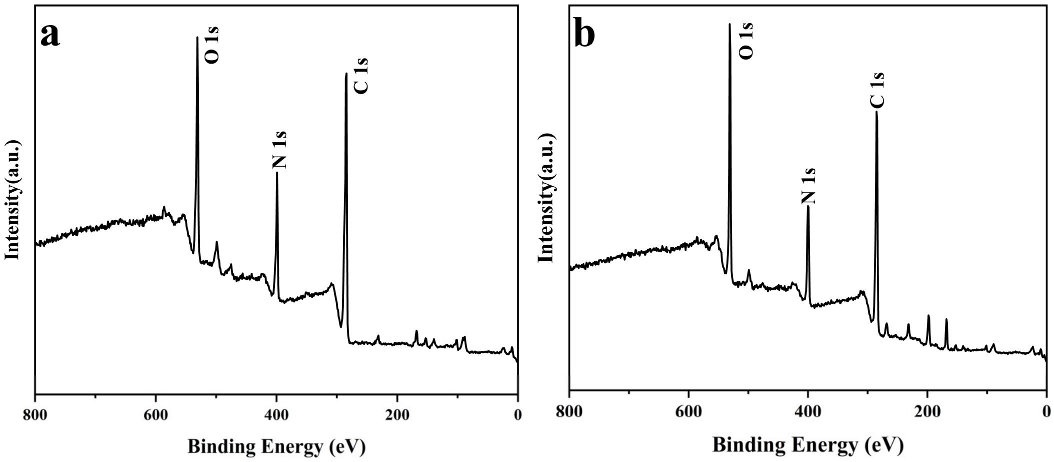


**Figure S2.** XPS wide scan spectrum of N-PAM **(a)** and p-(Amide)-PAM **(b)**.
